# Oesophageal perforation as a complication of ingested partial denture

**DOI:** 10.1259/bjrcr.20150348

**Published:** 2016-11-02

**Authors:** Himansu Shekhar Mohanty, Kapil Shirodkar, Aruna R Patil, Govindrajan Mallarajapatna, Sharath Kumar, K Chinmay Deepak, Shrivalli Nandikoor

**Affiliations:** Department of Radiology, Apollo Hospital, Bangalore, India

## Abstract

We report herein the case of a 53-year-old female who came to the emergency room with the chief complaints of severe dysphagia and chest pain following accidental swallowing of her denture. The patient had swelling of the face, neck and eyelids with difficulty in breathing. A skull radiograph was taken, which revealed a missing partial denture from the right lower jaw. Anteroposterior radiograph of the chest showed two metallic objects in the mid-thorax, adjacent to the descending aorta. CT scan of the neck and chest revealed two metallic objects (measuring approximately 17mm each) in the middle one-third of the oesophagus (right posterolateral aspect), causing perforation of the oesophagus and leading to pneumomediastinum, and left pneumothorax with subcutaneous emphysema of the neck and chest. An emergency thoracoscopic removal of the foreign body (partial denture) was performed with subsequent repair of the oesophageal tear in the same sitting. Post surgery, the patient was shifted to intensive care unit and she recovered well over a course of time. In summary, accidental ingestion of a partial denture can lead to grave complications such as oesophageal perforation, which should be managed on an emergency basis with thoracoscopic removal of the foreign body.

## Background

Accidental ingestion or aspiration of foreign bodies (FBs) is commonly seen in the extremes of age (children and elderly) and in mentally and physically debilitated patients. The most common accidental FB ingestion in the elderly includes loosely fitted dentures, fish bones and meat boluses.^1^ The ingested FB is likely to be lodged at points of narrowing in the oesophagus, which includes the cricopharynx, the aortic arch indentation, the left main bronchus indentation and the lower oesophageal sphincter. Once the FB has passed beyond the cricopharynx, it frequently remains in the oesophagus owing to weak peristalsis and multiple anatomically narrow areas.^2^ Sharp FBs such as dentures can cause oesophageal perforation and mediastinitis if overlooked.

In our case, the patient came to the emergency room (ER) within a few hours of ingestion of the partial denture with severe chest pain, dysphagia, odynophagia and breathlessness. The diagnosis of impacted FB causing oesophageal perforation with pneumomediastinum, pneumothorax and surgical emphysema was made with the help of radiography and CT imaging. Thoracoscopic removal was considered the ideal procedure in this case.^3^ The post-operative period was uneventful and the patient recovered well.

## Case presentation

A 53-year-old female presented to the ER with chief complaints of dysphagia, odynophagia, breathlessness and chest pain. She gave a history of accidentally swallowing some FB while taking her regular medication, which was now giving her a sensation of something stuck in her neck. On examination, her neck, face and eyelids were swollen, and she had subcutaneous crepitus on palpation. She had a dental repair performed 16 years ago, with metallic dentures fitted in both upper and lower jaws.

**Figure 1.**
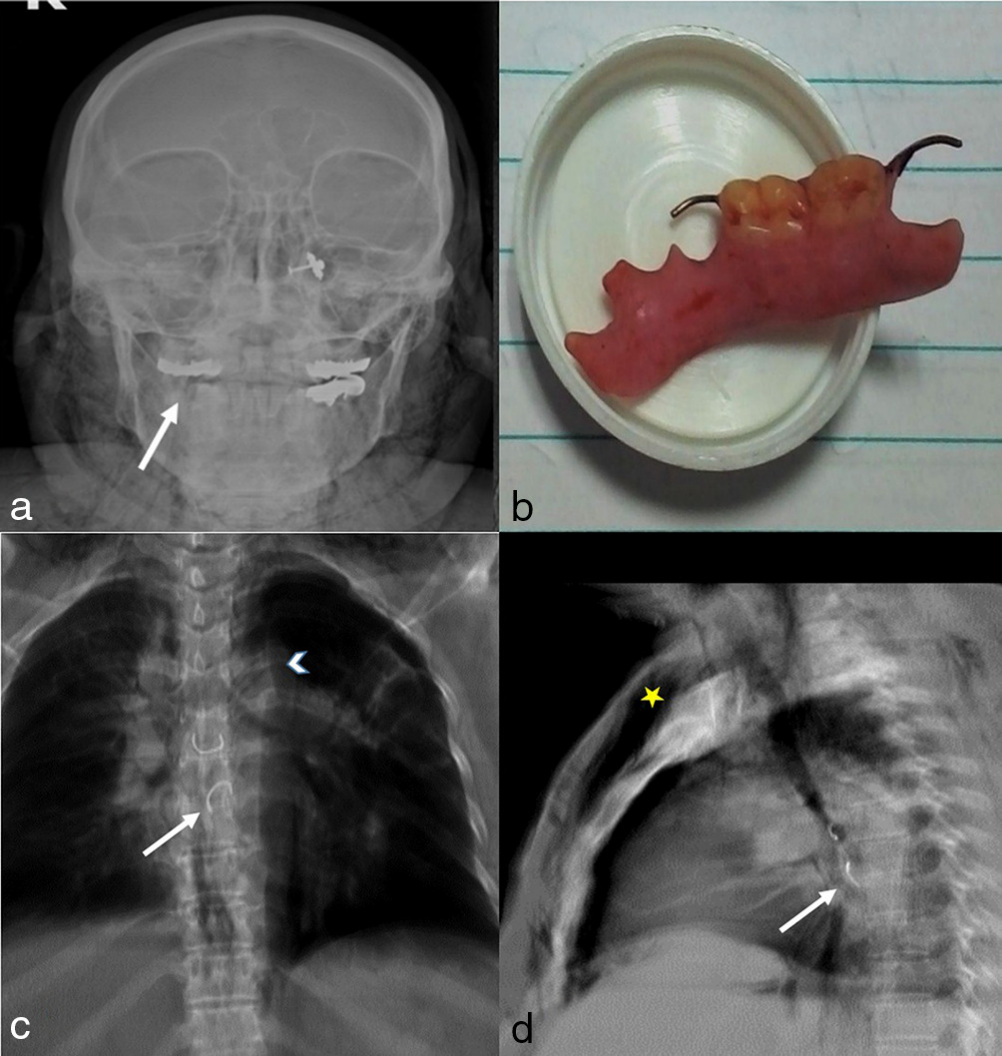
(a) Skull radiograph, frontal view, showing the missing denture from the right lower jaw (white arrow). (b) Thoracoscopically removed partial denture. (c, d) Anteroposterior and lateral view chest radiographs showing a metallic density foreign body in the retrocardiac region (white arrows), pneumomediastinum (arrowhead) and subcutaneous emphysema (yellow star).

A skull radiograph was taken, which showed a missing partial denture from the right lower jaw (Figure 1a).

Anteroposterior and lateral chest radiographs (Figure 1c,d) showed two metallic density objects in the retrocardiac area adjacent to the descending aorta with mild right-sided pleural effusion, pneumomediastinum and subcutaneous emphysema.

**Figure 2.**
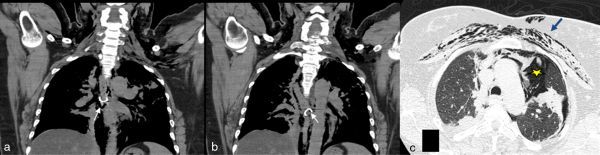
(a, b) Plain CT scan of the chest in coronal section showing a metallic density foreign body (white arrows) in the middle one-third of the oesophagus. (c) Axial non-contrast-enhanced CT scan showing pneumomediastinum (yellow star), subcutaneous emphysema (blue arrow) and left pneumothorax.

**Figure 3.**
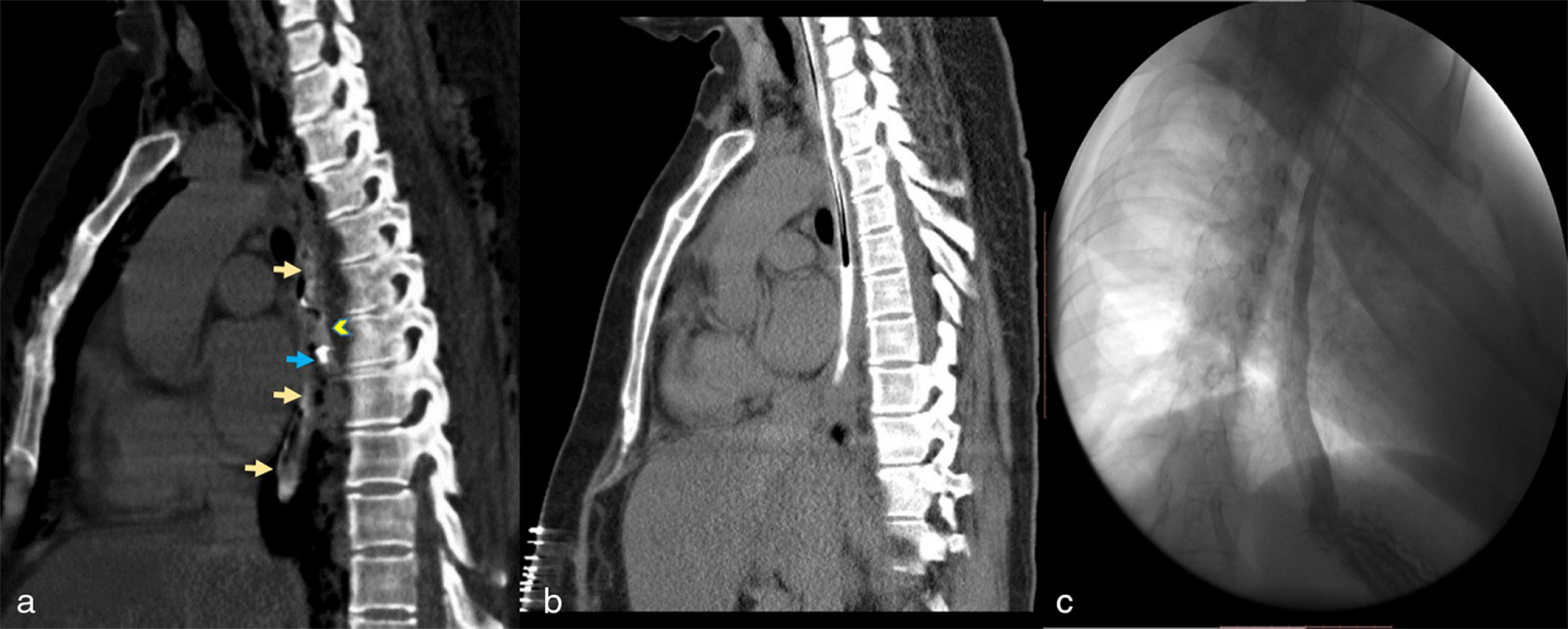
(a) Saggital plain CT scan of the chest with positive oral contrast; white arrows showing contrast column in the oesophagus; blue arrow showing metallic denture in the retrocardiac location; yellow arrowhead showing the leak of positive oral contrast at the site of the second denture fragment. (b, c) Post-operative seventh day plain coronal CT scan of the chest and gastrograffin study showing no leak in the repaired oesophagus with resolution of pneumothorax and subcutaneous emphysema.

Clinically, the suspicion of a perforated oesophagus was raised and CT imaging of the neck and thorax was ordered to confirm the diagnosis. On the CT scan, two metallic density objects (measuring approximately 17 mm each) were seen in the middle one-third of the oesophagus (Figure 2a,b), with a suspicious contained leak of orally ingested positive contrast media along the right posterolateral aspect of the oesophagus (Figure 3a). In addition, there was pneumomediastinum with left pneumothorax and subcutaneous emphysema of the neck and chest, which confirmed the diagnosis of oesophageal perforation secondary to ingested dentures (Figure 2c).

The patient was haemodynamically stable and was rushed for emergency thoracoscopic removal of the FB. Under thoracoscopic guidance, a rent of 2 cm and an ingested partial denture were confirmed in the right posterolateral aspect of the mid-oesophagus. The denture (Figure 1b) was removed and the oesophageal tear was repaired subsequently.

The patient was shifted to post-operative intensive care facility and started on broad-spectrum antibiotics. The post-operative period was uneventful. A follow-up oral gastrograffin study was performed on post-operative day 7, which revealed no leak. Per oral diet was then started gradually (Figure 3b,c).

## Discussion

FB ingestion is a common problem among children and the elderly population. FBs are most commonly ingested accidentally; however, incidences of non-accidental ingestion have also been cited in the literature. The most common FBs ingested by the elderly include fishbones or chickenbones, and dentures. Dentures are used for mastication and swallowing in the elderly, which providespleasant experience, and to enhance the clarity of speech, as well as improving facial aesthetics. With an increase in the denture-wearing population, the incidence of denture impaction has also increased.^4^ Following FB ingestion, patients usually present with dysphagia and odynophagia. Other symptoms include hypersalivation, retrosternal fullness and regurgitation of food. The diagnosis of FB ingestion is suspected if the patient gives a reliable history. Physical examination will reveal secondary signs of perforation such as subcutaneous emphysema or peritoneal signs. If there is obstruction of the bowel, drooling of saliva is another important secondary sign. Radiology can determine the exact site of a radiopaque-impacted FB.^3^ FBs can sometimes be demonstrated with plain radiographs of the neck or chest, or on conventional barium oesophagogram, but these procedures may fail to show FBs that are thin, small or only faintly radiopaque. CT scanning is considered superior to plain radiography in identifying FB in 70–100% of patients.^5–6^ A CT scan can demonstrate radiopaque FBs and exhibit superior images compared to plain radiographs, and visualize secondarily induced inflammatory changes in the adjacent structures suggesting the site of oesophageal perforation. Roura et al^7^ noted that 99% of ingested FBs become lodged in the upper gastrointestinal tract, with most of the FBs being found in the oesophagus. In some clinical situations, locating the FB through radiographic analysis becomes difficult as many materials used in dentistry, especially removable prostheses, have radiolucent characteristics.^8^

Eren et al^9^ in their study reported dental prosthesis as the FB in 3.1% of the cases. The management strategies for removal of FB include endoscopic removal, retrieval devices, thoracoscopic oesophagotomy and open surgery. Attempts at endoscopic removal of the dental prosthesis may cause intramural perforation or a full-thickness tear owing to possible entrapment of the wire hooks in the oesophageal wall. Thoracoscopic oesophagotomy represents a safe and effective treatment for patients with impacted dentures in the oesophagus.^3^

In our case, owing to the classical history, and extensive pneumomediastinum and subcutaneous emphysema on radiographs, oesophageal perforation was suspected early and was confirmed on the CT scan. The patient was taken quickly for thoracoscopic removal and secondary repair of the oesophagus. The post-operative recovery period was uneventful and the patient was discharged without significant morbidity.

Although oesophageal rupture secondary to ingested denture may not be uncommon, the unique feature of our case is the promptness of diagnosis of perforation visualized on the skull and chest radiographs, leading to shortening of the time interval between ER admission and surgery (approximately 4 h). This resulted in early recovery and reduced morbidity. Also, in this case, thoracotomy was performed instead of endoscopic retrieval, which also led to less post-operative complications.

## Learning points

1. History of sticking sensation in the neck should raise the suspicion of an impacted FB.
2. Drooling of saliva is an important secondary sign when obstruction of the oesophagus is suspected.
3. Classical history, subcutaneous emphysema, pneumomediastinum, pneumothorax and pleural effusion should raise the suspicion of oesophageal perforation.
4. Attempts at endoscopic removal of the dental prosthesis may cause intramural perforation or a full-thickness tear.
5. Thoracoscopic oesophagotomy represents a safe and effective treatment for patients with impacted dentures.

## Consent

Informed consent has been taken.
